# Repurposing Heparin as Antimalarial: Evaluation of Multiple Modifications Toward In Vivo Application

**DOI:** 10.3390/pharmaceutics12090825

**Published:** 2020-08-29

**Authors:** Elena Lantero, Carlos Raúl Aláez-Versón, Pilar Romero, Teresa Sierra, Xavier Fernàndez-Busquets

**Affiliations:** 1 Barcelona Institute for Global Health (ISGlobal, Hospital Clínic-Universitat de Barcelona), Rosselló 149-153, ES-08036 Barcelona, Spain; elantero@ibecbarcelona.eu; 2 Nanomalaria Group, Institute for Bioengineering of Catalonia (IBEC), The Barcelona Institute of Science and Technology, Baldiri Reixac 10-12, ES-08028 Barcelona, Spain; 3 BIOIBERICA S.A.U., Polígon Industrial “Mas Puigvert”, Ctra. N-II, km. 680.6, ES-08389 Palafolls, Spain; cralaez@bioiberica.com; 4 Instituto de Nanociencia y Materiales de Aragón (INMA), University of Zaragoza-CSIC, Pedro Cerbuna 12, ES-50009 Zaragoza, Spain; promero@unizar.es (P.R.); tsierra@unizar.es (T.S.); 5 Nanoscience and Nanotechnology Institute (IN2UB), University of Barcelona, Martí i Franquès 1, ES-08028 Barcelona, Spain

**Keywords:** malaria, heparin, *Plasmodium falciparum*

## Abstract

Heparin is a promising antimalarial drug due to its activity in inhibiting *Plasmodium* invasion of red blood cells and to the lack of resistance evolution by the parasite against it, but its potent anticoagulant activity is preventing the advance of heparin along the clinical pipeline. We have determined, in in vitro *Plasmodium falciparum* cultures, the antimalarial activity of heparin-derived structures of different origins and sizes, to obtain formulations having a good balance of in vitro safety (neither cytotoxic nor hemolytic), low anticoagulant activity (≤23 IU/mL according to activated partial thromboplastin time assays), and not too low antimalarial activity (IC50 at least around 100 µg/mL). This led to the selection of five chemically modified heparins according to the parameters explored, i.e., chain length, sulfation degree and position, and glycol-split, and whose in vivo toxicity indicated their safety for mice up to an intravenous dose of 320 mg/kg. The in vivo antimalarial activity of the selected formulations was poor as a consequence of their short blood half-life. The covalent crosslinking of heparin onto the surface of polyethylene glycol-containing liposomes did not affect its antimalarial activity in vitro and provided higher initial plasma concentrations, although it did not increase mean circulation time. Finding a suitable nanocarrier to impart long blood residence times to the modified heparins described here will be the next step toward new heparin-based antimalarial strategies.

## 1. Introduction

Despite being preventable and treatable, malaria continues to have a devastating impact on people’s health and livelihoods around the world. According to the last World Malaria Report [1], around 228 million cases of malaria occurred globally in 2018 (up from 216 million in 2016), and the disease led to an estimated 405,000 deaths, mostly children under five years of age in sub-Saharan Africa. Although there were an estimated 11 million fewer malaria cases in 2018 than in 2010, data for the period 2015–2018 highlight that no significant progress in reducing global malaria incidence was made in this timeframe. In the Greater Mekong subregion, there is now *Plasmodium falciparum* resistance to artemisinin and other drugs, which is leading to treatment failure. Resistance to antimalarial drugs has had a significant impact on the cost of global malaria control, as new drugs have had to be developed to replace those that have become ineffective. In this context, the application of the 3 Rs of drug development (rescue, repurpose, reposition) to previously discarded compounds is an interesting strategy to return value to potential treatments in decline or on hold.

Red blood cells (RBCs) infected with mature stages of the malaria parasite bind to the endothelial cells in the capillaries of tissues in a phenomenon known as sequestration, which allows *Plasmodium* to replicate while evading splenic clearance [2]. *Plasmodium*-infected RBCs (pRBCs) can also adhere to non-infected erythrocytes giving rise to rosettes, and they can form clumps through platelet-mediated binding to other pRBCs [3]. These events, which may lead to occlusion of the microvasculature, are thought to play a major role in the fatal outcome of severe malaria. Because the blood-stage infection is responsible for all symptoms and pathologies of malaria, pRBCs have traditionally been a main chemotherapeutic target [4]. One of the main pRBC-binding molecules are glycosaminoglycans (GAGs), a family of ubiquitous polysaccharides, some of whose members count among the most negatively charged natural polymers. Binding to the GAG chondroitin 4-sulfate (CSA) is thought to cause pRBC sequestration in the placenta, which has been linked to the severe disease outcome of pregnancy-associated malaria [5]. Heparan sulfate (HS), or a HS-like molecule exposed on RBCs, is the ligand responsible for rosetting [6], and is also targeted by the circumsporozoite protein in the sporozoite attachment to hepatocytes during the primary stage of malaria infection in the liver [7,8]. GAG-based therapies against malaria have been proposed in the wake of the results from different assays showing that soluble CSA, heparin, HS, heparin/HS derivatives, and other sulfated glycoconjugates can inhibit pRBC sequestration, disrupt rosettes, and block sporozoite adhesion to hepatocytes [9,10,11]. Heparin had actually been used in the treatment of severe malaria [12], but it was abandoned because of its strong anticoagulant action, with side effects such as intracranial bleeding. However, depolymerized heparin lacking anticoagulant activity has been found to disrupt rosette formation and pRBC cytoadherence in vitro and in vivo in animal models and in fresh parasite isolates [13].

Heparin has also a direct antimalarial activity on the pathogen, which operates through the inhibition of parasite invasion of RBCs [14], mainly by interaction with the merozoite surface protein 1 (MSP1) [15] involved in the initial contact and reorientation of the *Plasmodium* cell pursuing invasion [16]. Single-molecule force spectroscopy data have revealed a complete specificity of adhesion of heparin to late form pRBCs (schizonts) vs. RBCs, with a binding strength matching that of antibody-antigen interactions [17]. Confocal fluorescence analysis showed that when added to living pRBC cultures fluorescein-labeled heparin enters late schizonts about to burst and in only 15 min colocalizes with the intracellular parasites [18]. In agreement with this binding to the intraerythrocytic late stage pathogen, heparin has been described to inhibit the egress of merozoites from the parasitized RBC following its binding to MSP1 and to proteins found in the inner part of the RBC membrane [19]. Commercial heparin with a nominal mean molecular weight of 13,000 Da inhibits the in vitro growth of *P. falciparum* with an IC50 around 10 µg/mL (roughly 1 µM). Because heparin is eventually found in the blood, *Plasmodium* must have been exposed to it during its long coevolutionary history with humans and yet parasite resistance has not been described so far [15]. Several heparin modifications with reduced anticoagulant activity but maintaining significant antimalarial activity in vitro have been identified to have potential for novel drug development [20]. Of importance for an optimal inhibitory activity are the presence of *N*- and *O*-sulfate residues and of ≥2 sulfate units per disaccharide, specific spatial arrangements of sulfation requiring sulfate groups positioned together on a single saccharide unit, and a minimum chain length of six monosaccharide residues [15]. Overall, longer-chain heparin molecules of molecular weight >3 to 25 kDa showed a trend toward having higher inhibitory activity than shorter-chain forms <3 kDa [20]. Periodate oxidation of non-sulfated uronic acid residues, which has been reported to abolish anticoagulation [21], increased the activity of some compounds [20].

Here we have explored different combinations of heparin modifications such as chain length, sulfation degree and position, and glycol-split, with the objective of identifying heparin-derived structures having a reduced anticoagulant activity, but maintaining a significant antimalarial potency.

## 2. Materials and Methods

Except where otherwise indicated, reagents were purchased from Sigma-Aldrich Corporation (St. Louis, MO, USA), and reactions were performed at room temperature (22 to 24 °C). The lipids (all ≥99% purity according to thin layer chromatography analysis) 1,2-dioleoyl-*sn*-glycero-3-phosphocholine (DOPC), 1,2-dioleoyl-*sn*-glycero-3-phosphoethanolamine (DOPE), 1,2-dioleoyl-3-trimethylammonium-propane (DOTAP), 1,2-distearoyl-*sn*-glycero-3-phosphoethanolamine-*N*-[methoxy(polyethylene glycol)-2000] (DSPE-PEG), 1,2-dioleoyl-*sn*-glycero-3-phosphoethanolamine-*N*-(lissamine rhodamine B sulfonyl) (DOPE-Rho), and cholesterol were purchased from Avanti Polar Lipids Inc. (Alabaster, AL, USA) and stored at −20 °C.

### 2.1. Heparin Modification and Characterization

Heparin and dermatan sulfate were obtained from animal mucosae and chondroitin sulfate from animal cartilage following standard industrial manufacturing procedures. Unfractionated heparin (UH, >12 kDa) was modified by depolymerization, desulfation, oversulfation, conjugation to primaquine (PQ), and glycol-split, and for the molecules with more than one modification, those were applied in this order. All modifications were performed as described elsewhere [22], and are succinctly described below.

For the depolymerization by nitrous acid [23,24], 4 g of heparin were dissolved in 65 mL of H_2_O and cooled to 4 °C. After adding 75 mg of NaNO_2_, the pH was adjusted to 2 with 0.1 M HCl. The solution was stirred at 4 °C for 20 min, and then the pH was brought to 7.0 by addition of 0.1 M NaOH. 1 g of NaBH_4_ was added in several portions under stirring. After 2–3 h, the pH was adjusted to 4 with 0.1 M HCl, and 15 min later the solution was neutralized with 0.1 M NaOH. The products, medium molecular weight heparin (MMWH, 8 to 12 kDa), low molecular weight heparin (LMWH, 4–8 kDa), and ultralow molecular weight heparin (ULMWH, ≤4 Kda) were precipitated with three volumes of ethanol, then dissolved in water and recovered by freeze-drying.

For 2-*O*-desulfation [22], 500 mg of heparin were dissolved in 10 mL of 1 M NaOH and then heated at 85 °C for 1 h. After cooling below 30 °C, the solution was brought to pH 7 with 0.1 M HCl and heated at 70 °C for 48 h. Then, the samples were cooled, dialyzed against H_2_O (cellulose acetate membranes, 1000-Da cut-off), and recovered by freeze-drying.

For 6-*O*-desulfation [25], 200 mg of sodium heparin salt was passed through a column of Amberlite IR-120, neutralized with pyridine, and lyophilized to obtain pyridinium heparin salt, which was solubilized in 20 mL of dry pryridine, to which 4 mL of *N*,*O*-bis(trimethylsilyl)acetamide were added. The mixture was incubated 2 h at 60 °C until a clear solution was obtained. The reaction was terminated by adding 20 mL of water and the sample was dialyzed against H_2_O, its pH adjusted above 7 with NaOH, and dialyzed again immediately. The product was recovered by freeze-drying.

For *N*-desulfation and *N*-acetylation [22], pyridinium heparin salt, as previously obtained [25], was stirred at 20–25 °C in Me_2_SO:water (9:1) for 120 min to obtain molecules with *N*-desulfation. For obtaining *N*-acetylation, the previous compounds were incubated with acetic anhydride in alkaline aqueous medium (NaHCO_3_, 4 °C, 2 h). At the point of *N*-desulfation or *N*-acetylation, products were dialyzed against H_2_O and recovered by freeze-drying.

For oversulfation, to obtain a highly sulfated heparin, the procedure described by Maruyama et al. [26] was applied. Briefly, 100 mg of sodium heparin salt were subjected to cation-exchange chromatography to obtain tributylamine salt, lyophilized, and dissolved in 0.8 mL of *N*,*N*-dimethylformamide, which contained an excess of pyridine-sulfur trioxide. After 1 h at 40 °C, 1.6 mL of water were added, and the product was precipitated with three volumes of cold ethanol saturated with anhydrous sodium acetate and collected by centrifugation. The product was dissolved in water, dialyzed and recovered by freeze-drying. The resulting SO_3_^−^/COO^−^ (i.e., SO_3_^−^/disaccharide) ratio (see below for its determination) of oversulfated heparin was 3.0 (as compared to 1.9–2.0 for native UH).

Glycol-split was done by exhaustive periodate oxidation and borohydride reduction of UH or of a previously depolymerized and/or 2-*O*-desulfated sample [27]. In the first protocol, 250-mg samples were dissolved in 6 mL of H_2_O, and 6 mL of 0.1 M NaIO_4_ were added. After stirring the solution at 4 °C for 16 h in the dark, 1 mL of ethylene glycol was added to stop the reaction, and the solutions were dialyzed against H_2_O for 16 h. Solid sodium borohydride (60 mg) was added to the retentate solutions in several portions under stirring. After 2–3 h, the pH was adjusted to 4 with 0.1 M HCl, and after stirring for 15 min, the solutions were neutralized with 0.1 M NaOH. After desalting and a second dialysis against H_2_O, the final products were recovered by freeze-drying. In the second protocol, 250-mg samples were dissolved in 5 mL of 1 M NaOH and then heated at 60 °C for 30 min. After cooling below 30 °C, the solutions were brought to pH 7 with 0.1 M HCl and heated at 70 °C for 48 h to induce the partial conversion of iduronic acid (IdoA)_2_SO_3_ to galacturonic acid. After cooling and dialyzing against H_2_O, the product was recovered by freeze-drying.

PQ conjugation to MMWH: 2.0 g of MMWH were dissolved in 50 mL of deionized water and the pH of the solution was adjusted to 7.0. 1.35 g of PQ phosphate and 0.075 g of sodium cyanoborohydride were added and the reaction was stirred for 15 min at room temperature. pH was adjusted to 7.0 and stirring was continued for 24 h. Then, an additional 0.075 g of sodium cyanoborohydride were added and the reaction continued for another 24 h. The crude product was centrifuged at 5000 rpm for 15 min, and the supernatant was dialyzed against water until the permeate was colorless. 50 g of VOPC1074 resin were added to the retentate and the solution was stirred for 15 min. Supernatant was discarded and the resin was washed with 400 mL of deionized water for 1.5 h. The resin was filtered and treated with 250 mL of 15% (*w*/*v*) NaCl. The mixture was further stirred for 12 h at room temperature and then vacuum filtered. The filtrate was dialyzed again as before until chloride anions were not detected in the permeate. The product was recovered by freeze-drying.

The molecular weight of most samples was determined by high-performance size exclusion chromatography combined with triple detector array (HP-SEC/TDA) [28]; samples were dissolved at 20 mg/mL in 0.1 M NaNO_3_, and 100 µL were injected in the SEC/TDA equipment (Viscotek GPCmax with Viscotek module 305 TDA (Malvern Instruments Ltd., Malvern, UK). SO_3_^−^/COO^−^ ratio [29], anti-Xa factor [30], and activated partial thromboplastin time (aPTT) activity on dry basis [18] were determined according to established protocols.

### 2.2. NMR Experimental Procedure

NMR analysis was performed on a Bruker AVANCE 500 spectrometer operating at a frequency of 500.13 MHz for ^1^H and 125.75 MHz for ^13^C equipped with a 5 mm TBO probe. Spectra were processed with Bruker Topspin software version 3.6.2. Around 30–40 mg of heparin samples were dissolved in 0.4 mL of D_2_O and the samples were held at a temperature of 298 K during data acquisition. Samples were analyzed by 1D and 2D NMR spectroscopy. Heteronuclear single-quantum coherence (HSQC), proton–proton correlation spectroscopy (^1^H-^1^H COSY) and total correlation spectroscopy (^1^H-^1^H TOCSY) were used to characterize their structures. Chemical shift values were measured downfield from trimethylsilylpropionate sodium salt (TSP) as standard. The ^1^H-^1^H TOCSY spectra were run using 32 scans per t1 increment (400 points) and a mixing time of 80 ms. ^1^H-^13^C HSQC spectra were recorded with carbon decoupling during acquisition with 512 increments of 32 scans for each experiment. Two-dimensional diffusion ordered spectroscopy (DOSY) experiments were performed using stimulated echo sequence with bipolar gradient pulses [31]. Diffusion time (Δ) was set within the interval 220–320 ms. The pulsed gradients were incremented from 2% to 95% of the maximum strength in 16 spaced steps with a duration (δ) of 4–8 ms. (For the internal reference TPS these values were Δ = 140 ms and δ = 2.8 ms). The 2D plots show diffusion coefficient values D in [m^2^/s]. The NMR spectra of the heparin formulations selected for in vivo assays are presented in the Supplementary Materials.

### 2.3. P. falciparum Culture and Growth Inhibition Assays

*P. falciparum* 3D7 parasites (Malaria Research and Reference Reagent Resource Center, MR4) were cultured at 4% parasitemia and 3% hematocrit in a hypoxia incubator (cell culture CO_2_ incubator, ESCO, Singapore) with a 92.5% N_2_, 5.5% CO_2_, and 2% O_2_ gas mixture, using complete Roswell Park Memorial Institute (RPMI) 1640 medium (supplemented with 2 mM l-glutamine, 50 μM hypoxanthine, 5 g/L Albumax II, 25 mM HEPES, pH 7.2). A modification of this culture medium substituting Albumax II by 10% human inactivated plasma was also used when indicated. Serial dilutions in complete RPMI of each compound tested were incubated with *P. falciparum* 3D7 cells at 1% pRBC and 3% hematocrit in a final volume of 200 µL in 96-well plates (SPL Life Sciences Co., Ltd., Gyeonggi-do, Korea). Every dilution was prepared in triplicate, and the parasites were incubated under hypoxia for 44 h, when parasitemia was determined by flow cytometry, using either FACSCalibur or LSRFortesa (4 laser) cytometers (both from BD Biosciences, San Jose, CA, USA). For each cytometer, cell culture from each sample was diluted at either 0.024% or 0.03% hematocrit in phosphate buffered saline, pH 7.4 (PBS), containing 0.5 nM or 0.25 nM SYTO 11, respectively. Parasitemia percentage was recorded with BD FACSDiva (BD Biosciences) software and further analyzed with GraphPad Prism 6 software (GraphPad Software, San Diego, CA, USA). The experiments with the samples of interest for in vivo assays were repeated three times, twice with the parasites synchronized at ring stage through a 5% (*w*/*v*) sorbitol treatment [32], and once with the parasites synchronized at trophozoite stage through a 70% Percoll treatment [33]. The rest of samples were tested in ring stage synchronized cultures.

### 2.4. In Vitro Cytotoxicity Assays

Human umbilical vein endothelial cells (HUVECs American Type Culture Collection, Manassas, VA, USA) were cultured in a CO_2_ incubator using Medium 199 (M199, LabClinics, Barcelona, Spain) supplemented with penicillin-streptomycin (100 units and 0.1 mg/mL, respectively) and 10% fetal bovine serum (complete M199) in T-25 flasks (SPL Life Sciences Co., Ltd.), allowed to grow up to 70–80% convergence and replated by trypsin treatment. Unspecific toxicity of the samples was tested with the WST-1 cell viability assay (Roche Applied Science, Penzberg, Germany), following the manufacturer’s recommendations. Briefly, HUVECs were seeded in 96-well plates at a density of 5000 cells per well in 100 µL of complete M199. After a 24-h incubation at 37 °C, the medium was removed, and 90 µL of fresh M199 were added together with 10 µL of the sample of interest in PBS. HUVECs were placed back in the incubator for 24 h or 48 h. At the moment of reading, 10 µL of WST-1 reagent was added to each well, and, after an incubation of 3–4 h, absorbance at 440 nm was measured with an Epoch^TM^ microplate spectrophotometer (BioTek Instruments Inc., Winooski, VT, USA). For each sample, three different concentrations were tested in triplicates, and each plate contained three seeded wells with 1% bleach (0% viability control) and three wells with 10 µL PBS (100% viability control) as controls.

### 2.5. Hemolysis Assays

In a 96-well plate, 2 µL of sample were added to 200 µL of a 3% hematocrit RBC suspension in RPMI complete medium. After incubating for 3 h at 37 °C, samples were centrifuged at 1000× *g* for 5 min and 150 µL of supernatant from each sample was transferred to a new plate where absorbance was measured at 541 nm in an Epoch^TM^ microplate spectrophotometer (BioTek Instruments Inc.). Assays were done in triplicates, including positive (Triton X-100) and negative (PBS) controls. Data analysis was done with Excel and GraphPad Prism 6 software.

### 2.6. In Vivo Toxicity Assays

Seven-week-old BALB/c female mice (18–20 g, Janvier Laboratories, Le Genest-Saint-Isle, France) were maintained with ad libitum access to food and water under standard environmental conditions (20–24 °C and 12 h/12 h light/dark cycle). The animals were anesthetized with isoflurane (4% for induction and 2.5% for maintenance) in an oxygen stream to ensure administration and minimize injection stress, while delivery of a 200 µL bolus was done intravenously. An adaptation of OECD 425 Test Guideline, which consisted of a single ordered dose progression, was followed in order to reduce the number of animals used. The first mouse received a dose one order of magnitude lower than the concentration proven safe in vitro, and the dose for the next animal was either decreased or increased by a factor of 3.2 depending on the observation or not, respectively, of acute effects on the first animal. After administration, each mouse was monitored for at least 48 h before the next animal was treated. In addition, other toxicity signs were evaluated maintaining all animals under observation for 14 days after dose injection. Following this protocol, different GAG concentrations (31.5, 100, 320, and 750 mg/kg) were tested, prepared in PBS from a 50 mg/mL stock solution of the compound in sterile PBS. When any toxic effects were observed, including, among others, >20% reduction in weight, aggressive and unexpected behavior or the presence of blood in faeces, animals were immediately anesthetized using a 100 mg/kg Ketolar plus 5 mg/kg Midazolan mixture and sacrificed by cervical dislocation. The highest dosage exhibiting absence of toxicity signs was considered the compound maximum tolerated dose. The animal care and use protocols followed adhered to the specific national and international guidelines specified in the Spanish Royal Decree 53/2013, which is based on the European regulation 2010/63/UE. The corresponding protocols were reviewed and approved by the Ethical Committee on Clinical Research from the Hospital Clínic de Barcelona (Reg. HCB/2018/1223, 23 January 2019).

### 2.7. Antimalarial Activity in Mice

A four-day suppressive test in BALB/c female mice was performed following pre-established protocols [34]. Briefly, animals were infected with 2 × 10^7^ pRBCs from a *Plasmodium yoelii yoelii* 17XL–infected mouse (20–30% parasitemia). Between 3 h and 4 h later, mice were treated intravenously with 100 µL of the test samples; an infection control group treated with PBS only and a treatment control group dosed with 5 mg/kg chloroquine were also included. For the next three days animals were treated following the same procedure at the same times. From day 2 post-infection, blood samples were collected by tail punction, and pRBC percentage was determined by either flow cytometry or blood smear preparation stained with Giemsa followed by optical microscope analysis.

### 2.8. Preparation of Heparin-Coated Liposomes

The lipid formulation DOPC:DOPE:cholesterol:DOTAP:DSPE-PEG:DOPE-Rho 46.5:30:20:2:2:0.5 was obtained by mixing stock solutions of lipids in chloroform in a round bottom flask. The solvent was evaporated by N_2_ flow and the lipid film was further dried under vacuum for 1 h. Then, lipids were hydrated in 1 mL of PBS and vortexed for 3 min, to achieve a final total lipid concentration of 20 mM. To obtain unilamelar liposomes of regular size, the suspension was extruded through 200 nm polycarbonate membranes (Avanti Polar Lipids, Inc.) using a mini extruder device (Avanti Polar Lipids, Inc.). UH at 20 mg/mL was activated for 30 min in 25 mM 2-(*N*-morpholino)ethanesulfonic acid, pH 5, with 39 mM 1-ethyl-3-(3-dimethylaminopropyl)carbodiimide (BioRad) and 55 mM *N*-hydroxysulfosuccinimide. Then, 500 µL of activated UH were added to a liposome suspension containing 0.67 mM total lipid in 3 mL of PBS and incubated under stirring for 2 h. To remove unbound heparin, the sample was centrifuged in a 100 kDa cut-off Amicon^®^ Ultra centrifugal filter and PBS was added to recover the initial volume; this process was repeated five times, until heparin was not detected in the recovered washes according to Alcian Blue quantification [35]. Heparin concentration in the final UH-coated liposome sample was 1.7 mg/mL, corresponding to a ca. 1:15 UH:total lipid molar ratio. Liposomes were detected in plasma by rhodamine fluorescence detection in an Infinite^®^ M Nano microplate reader spectrofluorometer (Tecan, Männedorf, Switzerland) at 553 nm excitation and 586 nm emission.

### 2.9. Plasma Half-Life Determination

BALB/c mice were inoculated intravenously with 18 mg/kg of the compounds to be tested (UH, 2-*O*-desulfated glycol-split MMWH, or UH-coated liposomes). Blood was collected at different times after administration via facial vein or cava vein extraction under isoflurane anesthesia. Collected blood was mixed with 1/10 volume of 3.2% sodium citrate, and plasma was separated by centrifugation (500× *g*) and frozen until quantification with the Heparin Red^®^ method (Redprobes UG, Münster, Germany), following published protocols [36]. In brief, 20 µL of non-treated mouse plasma containing different heparin concentrations (30, 20, 15, 10, 7.5, 5, 2, 1, and 0 µg/mL) and of the collected plasma from treated mice were placed in duplicates per mouse and time point in a 96-well plate. Enhancer solution, 1 M MgCl_2_ and Heparin Red^®^ were mixed (85.5:4.5:1 for UH samples and 171:9:1 for 2-*O*-desulfated glycol-split MMWH), 80 µL of the mixture was added to each well, the plate was shaken for 3 min, and fluorescence was recorded at 590 nm excitation and 645 nm emission, using a Synergy microplate reader (BioTek Instruments Inc.).

### 2.10. Ethics Statement

The human blood and plasma used for *P. falciparum* in vitro cultures were commercially obtained from the *Banc de Sang i Teixits* (www.bancsang.net). Purchased units had been discarded for transfusion, mostly due to an excess of blood relative to anticoagulant solution. Prior to use, blood and plasma units underwent the analytical checks specified in the current legislation. Before being delivered, to guarantee the non-identification of the blood donor, unit data were anonymized and irreversibly dissociated, and any identification tag or label was removed. No blood data were or will be supplied, and the studies reported here were performed in accordance with the current Spanish *Ley Orgánica de Protección de Datos* and *Ley de Investigación Biomédica* and under protocols reviewed and approved by the Ethical Committee on Clinical Research from the *Hospital Clínic de Barcelona* (Reg. HCB/2018/1223, 23 January 2019).

## 3. Results

### 3.1. Antimalarial Activity Determination of Different Natural GAGs

**Figure 1:**
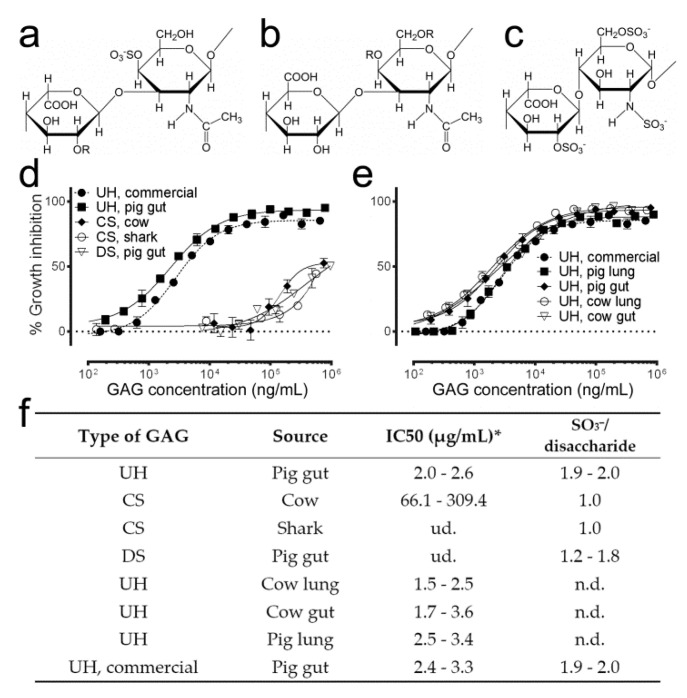
In vitro antimalarial activity of glycosaminoglycans (GAGs) from different origin. (**a**–**c**) Structures of the most represented disaccharide units in (**a**) dermatan sulfate, (**b**) chondroitin sulfate and (**c**) heparin. R: H or SO_3_^−^. (**d**,**e**) Graphs comparing the in vitro *Plasmodium falciparum* growth inhibition activity of different GAGs. (**f**) Description of the GAGs tested in panels (**d**,**e**). UH: unfractionated heparin, CS: chondroitin sulfate, DS: dermatan sulfate. ud.: undetected, n.d.: not determined. Commercial UH was purchased from Sigma Aldrich (Cat. No. H-4784). * IC50 has been calculated by non-linear regression of the percentage of growth inhibition against molecule concentration. IC50 range represents 95% confidence interval of one experiment.

Preliminary in vitro antimalarial activity assays of different GAG types (Figure 1a–c) were consistent with previously published data [20] indicating that a >30-fold higher amount of dermatan or chondroitin sulfate than that of heparin was required to obtain similar parasite growth inhibitions (Figure 1d–f). A higher sulfate content in heparin (1.9–2.0 sulfate groups/disaccharide) correlated with its higher antimalarial activity. The source from which heparin was obtained did not have a significant influence on its capacity to inhibit *P. falciparum* growth in vitro.

### 3.2. Effect on Antimalarial Activity of Heparin Molecular Weight

**Figure 2:**
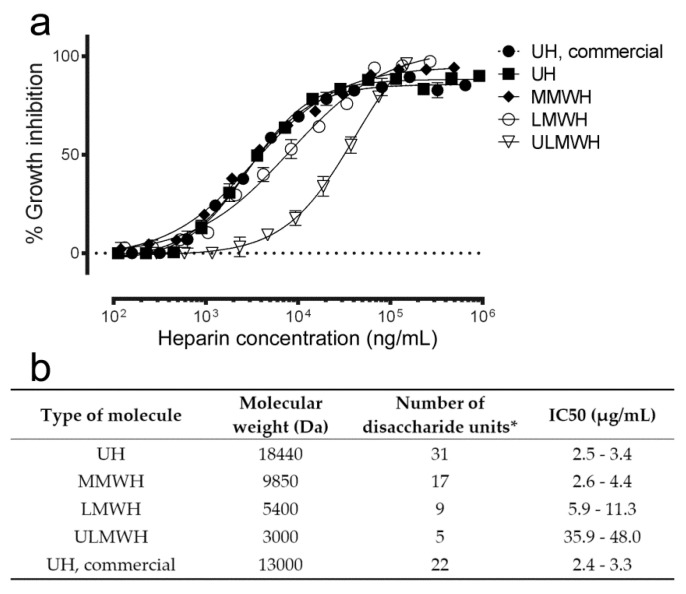
In vitro antimalarial activity of heparins with different molecular weight. (**a**) In vitro *P. falciparum* growth inhibition activity assay of heparin fractions of decreasing chain lengths. (**b**) Description of the heparin samples tested in panel (**a**). MMWH: medium molecular weight heparin, LMWH: low molecular weight heparin, ULMWH: ultralow molecular weight heparin. * The approximate number of disaccharide units was calculated considering the molecular weight of the trisulfated disaccharide unit as 590.9 g/mol. IC50 range represents 95% confidence interval of one experiment.

Because higher molecular weights of heparin are usually related with undesired secondary effects, such as induced thrombocytopenia or hemorrhage [37], in vitro parasite growth inhibition was determined for unfractionated heparin from pig lung and for different fractions obtained from this molecule as precursor. In agreement with previous reports [15], both the curves of percentage of growth inhibition and the derived IC50 values showed that shorter heparin chain lengths had a reduced antimalarial activity, especially for ULMWH (Figure 2). Decreasing the number of disaccharide units below nine significantly lowered the antiplasmodial activity of heparin in vitro.

### 3.3. Effect of Sulfate Group Removal on the Antimalarial Activity of Heparin

**Figure 3:**
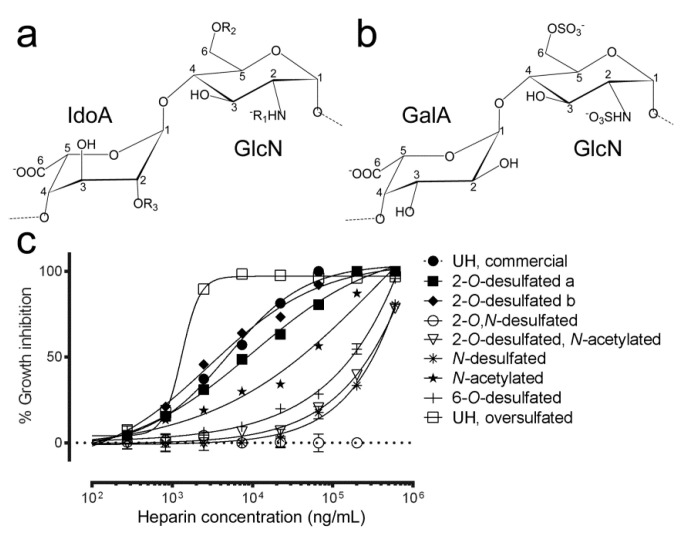
In vitro antimalarial activity of heparins with different sulfation patterns. (**a**,**b**) Structures obtained after desulfation (see Table 1 for the definition of chemical groups R_1_, R_2_, and R_3_). IdoA: iduronic acid, GlcN: glucosamine, GalA: galacturonic acid. (**c**) In vitro *P. falciparum* growth inhibition activity assay of heparin fractions with different sulfation patterns.

Because there is a direct correlation between the number and position of sulfate groups and the anticoagulant activity of heparin [38], the antimalarial capacity of different desulfated heparin structures was analyzed (Figure 3). 2-*O* desulfation of IdoA together with glucosamine *N*-desulfation completely suppressed antimalarial activity, whereas 6-*O*-desulfation and *N*-desulfation significantly increased the IC50 of the resulting structures to >300 µg/mL (Table 1).

2-*O*-desulfation of IdoA significantly reduced the anticoagulant activity of heparin according to aPTT values. This modification had only a moderate effect on antimalarial activity, except when the glucosamine *N*-sulfate was also removed or substituted by an acetyl group, which completely abolished the antiplasmodial action of heparin. The affinity for antithrombin of the two 2-*O*-desulfated samples maintaining a good inhibition of *Plasmodium* growth is likely suppressed, since anti-Xa activity was very low (3 IU/mg and 9 IU/mg for 2-*O*-desulfated structures in Figure 3 panels a and b, respectively) when compared to that of UH (192 IU/mg). The galacturonic acid (stereoisomer of IdoA) modification, shown in panel b in Figure 3, had an in vitro antimalarial activity that improved that of commercial UH. This inversion increases the chain rigidity [38], and it has been suggested that an increment in such rigidity of heparin-like molecules can produce an increase of antimalarial activity [15].

Oversulfated heparin exhibited an increased antimalarial activity relative to unfractionated heparin, although this positive result was not accompanied by a dramatic decrease in anticoagulant activity (94 IU/mg vs. 203 IU/mg, respectively).

### 3.4. Effect of Glycol-Split on the Antimalarial and Anticoagulant Activities of Heparin

**Figure 4:**
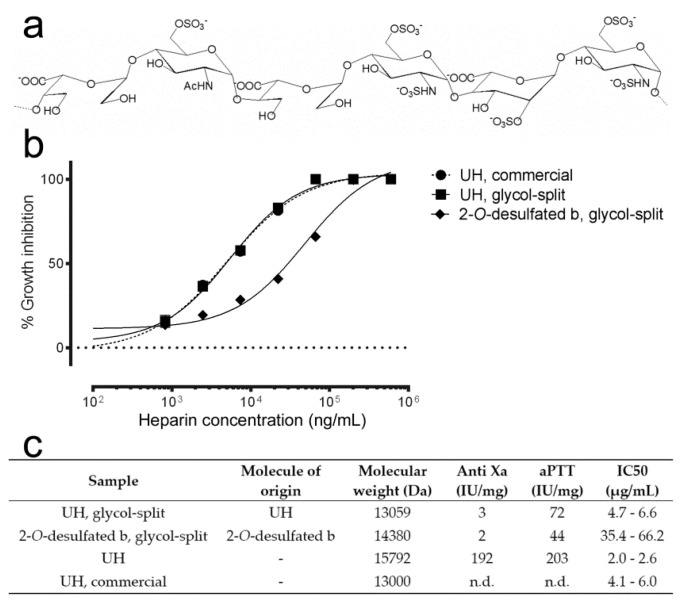
Effect of glycol-split on the in vitro antimalarial activity of heparin. (**a**) Structure of the heparin chain after glycol-split treatment. (**b**) In vitro *P. falciparum* growth inhibition activity assay of heparin fractions with and without glycol-split. (**c**) Description of the heparin samples tested in panel (**b**). n.d.: not determined.

Whereas a moderate degree of glycol-split (Figure 4a) slightly reduced the antimalarial activity of heparin (Figure 4b), it dramatically lowered its anticoagulation action according to the aPTT assay (72 IU/mg vs. 203 IU/mg relative to unfractionated heparin; Figure 4c). When glycol-split was applied to the 2-*O*-desulfated structure having the best balance between antimalarial and anticoagulant activities (Figure 3b), aPTT was further decreased (44 IU/mg), although this was accompanied by a decrease in the capacity to inhibit *Plasmodium* growth in vitro. This sample was derived from the 2-*O*-desulfated structure shown in Figure 3b, which had a higher degree of oxidation leading to increased glycol-split, and therefore to more open rings and flexibility than the sample derived from unfractionated heparin. Glycol-split treatment of heparin chains has been described to increase chain flexibility and to decrease interactions with coagulation factors [39], as evidenced by the dramatic decrease in antithrombin binding of glycol-split heparins (Figure 4c).

### 3.5. Selection of Heparin Forms Having Reduced Anticoagulant Activity but Maintaining Significant Plasmodium Growth Inhibition

To advance toward the use of heparin as a clinically useful antimalarial drug, keeping a low anticoagulant activity will be as important as maintaining its capacity to inhibit the growth of the parasite, and therefore a delicate balance has to be met between these two indicators. Several structures having less anticoagulant activity than unfractionated heparin were obtained when applying different combinations of the parameters explored above, i.e., chain length, sulfation degree and position, and glycol-split (Table 2). None of the resulting preparations exhibited significant hemolysis or unspecific toxicity in HUVEC cultures. Since heparins with reduced anticoagulant activity could be used in vivo in larger amounts, a sufficient antimalarial activity could be in principle obtained with them, even if they have a relatively low antiplasmodial potency.

### 3.6. Conjugation of Heparin with PQ

**Figure 5:**
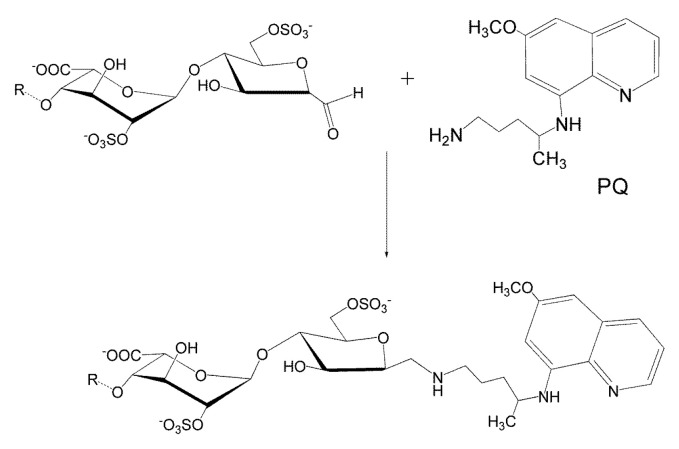
Reaction between the reducing end of heparin and primaquine (PQ), and the resulting molecular structure.

To explore alternative strategies that could compensate for the loss in antimalarial activity of modified heparins, the antimalarial drug PQ was conjugated to the reducing end of MMWH (Figure 5). The combination of both molecules resulted in an antimalarial potency that significantly improved that of MMWH alone (Table 3). This result could be interpreted as a targeting effect of heparin, whose known affinity for pRBCs [17,18] might contribute to a more efficient delivery of PQ to target cells. This approach offers a potential solution to recover part of the antimalarial activity of heparin, which is lost with the chemical modifications that confer it a reduced anticoagulant action.

### 3.7. Antimalarial Activity In Vivo of Heparin-derived Structures

For antimalarial in vivo assays were selected those heparin-derived structures that exhibited a good balance of in vitro safety (neither cytotoxicity nor hemolysis observed), low anticoagulant activity (≤23 IU/mL in aPTT assays) and not too low antimalarial activity (IC50 at least around 100 µg/mL). This led to the selection of the five heparins shadowed in grey in Table 2, whose in vivo toxicity was determined as an additional preliminary check. All five samples were not toxic when administered intravenously at 320 mg/kg (Table 4). Full characterization by NMR (Figures S1–S8) was performed to confirm presence of the expected modifications.

**Figure 6:**
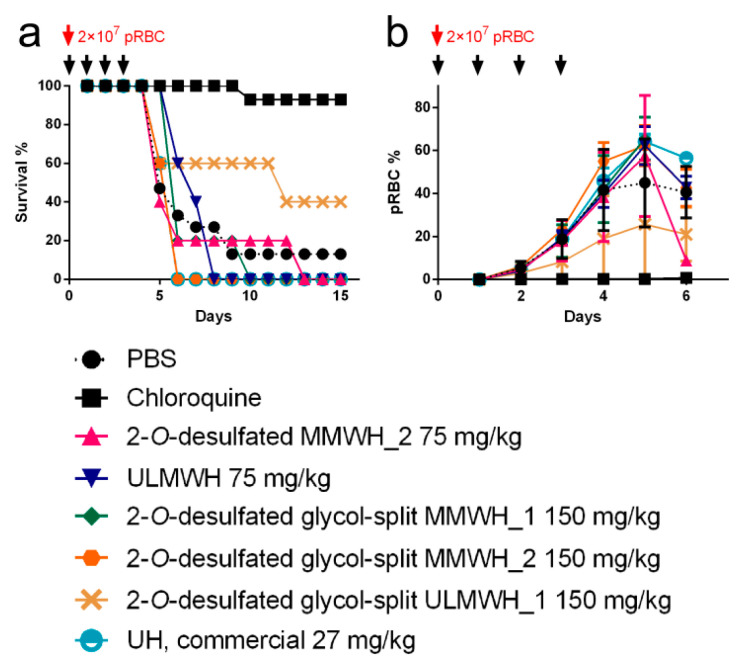
Antimalarial activity in vivo of heparin-derived structures. Mice survival curves (**a**) and parasitemia percentage (**b**) following treatment with the tested compounds at the concentrations indicated. The black arrows indicate the times of test sample administration. *n* = 5 in each group, except PBS- (*n* = 15) and chloroquine-treated mice (*n* = 14).

When the five selected heparin formulations were tested in vivo in the *P. yoelii yoelii* 17XL rodent malaria model by intravenous administration in a 4-day suppressive test, neither animal survival nor parasitemia load were significantly improved relative to the control PBS-treated group (Figure 6). Although a tendency was observed toward increased survival time of heparin-treated mice, this result could be due to small differences in parasite inoculation among different animals.

### 3.8. Determination of the Circulation Time of Intravenously Administered Heparin

**Figure 7:**
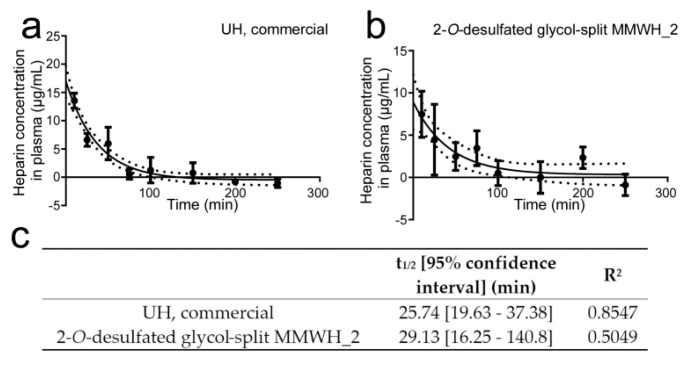
Determination of blood circulation time for unfractionated heparin (UH) and 2-*O*-desulfated glycol-split MMWH_2. (**a**,**b**) Concentration of both heparins in mouse plasma along time following intravenous administration to three male and three female mice for each time point. (**c**) Blood half-life values (t_1/2_) for both samples and R^2^ of the plotted curves.

**Figure 8:**
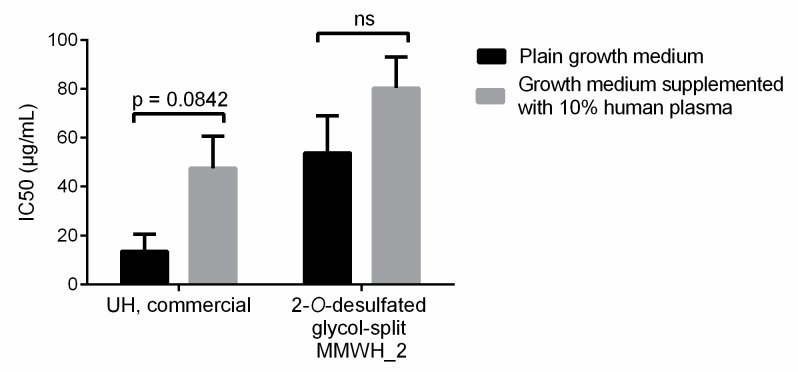
IC50 of the two samples from Figure 7 in *P. falciparum* in vitro cultures grown in either plain growth medium or in growth medium supplemented with 10% human plasma. ns: not significant.

The poor efficiency of the selected formulations for the treatment of in vivo infections could be due, among other reasons, to a rapid elimination from the blood circulation. Because the antimalarial activity of heparin resides in its inhibition of the red blood cell invasion by the merozoite form of the parasite, a phase that lasts only a few minutes within the *P. falciparum* 48-h intraerythrocytic life cycle, a rapid elimination from the blood circulation would significantly reduce the chances of being present when invasion takes place. Commercial UH and 2-*O*-desulfated glycol-split MMWH_2 had similar plasma half-lives (25.7 and 29.1 min, respectively; Figure 7) and became undetectable about 3 h after intravenous administration to mice, suggesting a fast clearance possibly due to interactions with plasma components. In addition, the in vitro activity of these two heparins became reduced when tested in growth medium supplemented with 10% human plasma (Figure 8), indicating that such plasma interactions might compete with the binding to merozoites of heparin, thus decreasing its antimalarial activity.

**Figure 9:**
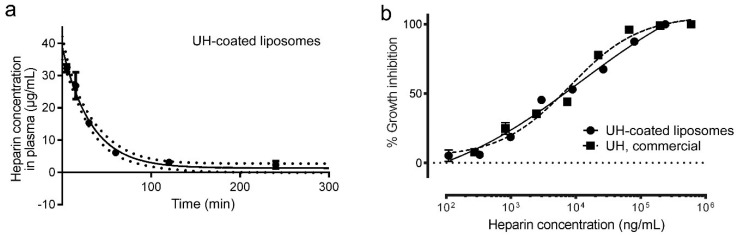
Characterization of circulation time and antimalarial activity of UH-coated liposomes. (**a**) Concentration of liposome-bound UH in mouse plasma along time following intravenous administration to 2 female mice for each time point. (**b**) *P. falciparum* growth inhibition activity assay of UH-coated liposomes and free UH.

To prevent the absorption of serum proteins, the surface of liposomes can be decorated by hydrophilic and bio-compatible polyethylene glycol (PEG) polymers, which can significantly extend blood circulation times [40]. We explored whether the covalent link of heparin to the surface of PEGylated liposomes increased its blood half-life and affected its antimalarial activity in vitro. The circulation half-life of UH-coated liposomes was 22.4 min (18.4–28.6 min with 95% confidence; Figure 9a), but they could be detected up to 4 h after administration (data not shown). The initial UH concentration 10 min after administration was ca. 40 µg/mL for UH-coated liposomes (as compared to ca. 17 µg/mL for free UH; Figure 7a), whereas the antimalarial activity in vitro of UH-covered liposomes had roughly the same IC50 as UH alone (Figure 9b). Although the liposomal formulation did not significantly extend the half-life of heparin in circulation, it did increase the time that the molecule was found in plasma at a higher concentration than its in vitro IC50 (21 min for free UH and 49 min for UH-coated liposomes).

## 4. Discussion

Previous works with sulfated GAGs, heparin modifications, and other sulfated compounds that could act as antimalarials [15,20,41] had placed this type of molecules as good candidates for the development of new drugs. Our data indicate that chemical modification of heparin might not be the only path to a GAG that could offer good perspectives of entering the clinical pipeline. Chondroitin sulfate has in vitro antimalarial activity comparable to those of the chemically desulfated structures prepared in this work, to which has to be added the benefit of a low anticoagulant activity [41]. Other sources of sulfated polysaccharides exhibiting antimalarial activity and inhibition of RBC invasion by *Plasmodium* at low anticoagulant concentrations are certain marine organisms such as algae, sea cucumbers and sponges [41]. Dermatan sulfate has also been explored to control *Plasmodium berghei* infections in mice, but although it could reduce parasitemia in the treated groups, there was no significant effect on animal survival [14]. These and other natural polysaccharides could be modified to improve their antimalarial capacity in a similar way as it was done with sevuparin, a heparin derivative that has reached phase I/II of clinical trials for malaria treatment as adjuvant therapy [42].

Besides their potential applications as antimalarial therapy through their blocking activity of RBC invasion by *Plasmodium* merozoites, the heparin modifications explored here could be of use for other pathological features of the disease. In severe malaria, pRBCs can adhere to naïve RBCs to form cell clumps termed rosettes, which can reduce the blood circulation in capillaries leading to life-threatening conditions [13]. Because heparin and other GAGs can disrupt rosettes, their administration as adjuvant therapy could reduce their formation. As previous research in this field has proven, the molecular structure of the GAG chains that interact with rosette forming pRBCs is variable and highly dependent on the particular *Plasmodium* strain [13]. In this regard, the implementation of a chemical modification toolbox including reactions like those presented in this work will be highly instrumental in the adaptation of these polysaccharides to their target cell types.

Of the different heparin modifications assayed in this work, a combination of reduction in size (MMWH), 2-*O*-desulfation of IdoA, and glycol-split has offered the best balance toward a structure having low anticoagulant activity but maintaining a still acceptable in vitro antimalarial action in *P. falciparum* cultures while showing low hemolysis and in vitro and in vivo toxicities. However, one of the main obstacles that we have identified on the way to a potential clinical application as a treatment for malaria is the fast clearance of heparin from the circulation. The blood half-life of exogenously administered heparin is dependent on the dose, and it can be cleared slowly through the renal system or through a fast but saturable mechanism that involves binding to cell receptors and macrophages [37]. Depolymerization of the heparin chain has been described to increase circulation time because it reduces some of the undesired interactions with cell surfaces [43]. Conjugation to nanocarriers also has the potential to impart longer blood residence times [44], and there is evidence of loss of the anticoagulant activity of heparin when covalently immobilized on a substrate [45]. Several types of nanostructures have been used as such substrates, e.g., liposomes [18], polymersomes [46], and giant unilamellar vesicles [47]. Heparin has been shown to work as pRBC targeting element of liposomes loaded with antimalarial drugs, which added to its invasion blocking activity in a peculiar type of combination therapy [18]. Heparin itself has been directly conjugated to the highly hydrophobic antimalarial drug artesunate to form micellar nanostructures with improved pharmacokinetic profile [48].

Heparin has an affordable production cost and its purification from animal tissues does not need complicated protocols or costly equipment. The endogenous nature of heparin makes it highly biocompatible and biosafe, especially in the case of low molecular weight heparin, whose chemical preparation is simple and inexpensive. Among the various materials available to form nanocarriers, natural polymers such as glycosaminoglycans are an ideal candidate due to their often endogenous, non-immunogenic nature, ease of availability in relatively large amounts at a comparatively low cost, and presence in their structures of adequate chemical groups for the use of straightforward crosslinking reactions into nanoparticulate structures. Chitosan, a positively charged glycosaminoglycan, is being extensively employed as drug carrier in many clinical applications already and therefore its adaptation to the transport of antimalarial drugs would be immediate. Heparin, a glycosaminoglycan whose antimalarial activity is well known, could be easily incorporated into chitosan nanocarriers, since its high negative charge will provide a strong interaction with chitosan, which if required can be strengthened through a simple chemical reaction by covalent bonds between the abundant amino and carboxyl groups present in chitosan and heparin, respectively. Heparin has been described to have binding affinity for several *Plasmodium* stages, in both human and mosquito hosts, thus being an interesting targeting element of therapeutic nanovessels. In addition to the antiparasitic and targeting activities of heparin, such hybrid glycosaminoglycan nanovectors can be loaded with drugs, sky-rocketing their potential activity against the pathogen. As an added bonus of using chitosan nanoparticles is that they are inexpensive and easy to produce, and are apt to be used in oral therapy [49,50].

## Figures and Tables

**Table 1:** Description of the heparin samples tested in Figure 3.

Sample	Molecule of Origin	Structure	Molecular Weight (Da)	aPTT (IU/mg)	IC50 (µg/mL)
2-*O*-desulfated structure a	UH	R_1_,R_2_: SO_3_^−^; R_3_: H	10,721	34	7.6–13.5
2-*O*-desulfated structure b	UH	b	14,023	63	2.2–4.1
2-*O*-desulfated, *N*-desulfated	2-*O*-desulfated a	R_1_,R_3_: H; R_2_: SO_3_^−^	13,459	20	ud.
2-*O*-desulfated, *N*-acetylated	2-*O*-desulfated a	R_1_: Ac; R_2_: SO_3_^−^; R_3_: H	10,868	24	>1000
*N*-desulfated	UH	R_2_,R_3_: SO_3_^−^; R_1_: H	15,771	42	>1000
*N*-acetylated	*N*-desulfated	R_2_,R_3_: SO_3_^−^; R_1:_ Ac	15,963	91	>300
6-*O*-desulfated	UH	R_1_,R_3_: SO_3_^−^; R_2_: H	14,521	45	>1000
UH	-	R_1_,R_2_,R_3_: SO_3_^−^	15,792	203	2.0–2.6
UH, oversulfated	UH	R_1_,R_2_,R_3_: SO_3_^−^	19,990	94	1.2–1.4
UH, commercial	-	R_1_,R_2_,R_3_: SO_3_^−^	13,000	n.d.	4.1–6.0

ud.: undetected, n.d.: not determined.

**Table 2:** Characterization of heparin samples that combine different chemical modifications. Shadowed in gray are the formulations selected for in vivo assays.

Sample	Mw (Da)	SO_3_^−^/Disaccharide	IC50 (µg/mL ± SD)	IC90 (µg/mL ± SD)	aPTT (IU/mg)	In Vitro Toxicity ^1^ (% ± SD)	Hemolysis ^1^ (% ± SD)
MMWH_1	10,497	n.d.	22.7 ± 2.4	178.1 ± 28.4	110	1.1 ± 3.4	0.1 ± 0.2
MMWH_2	11,000	1.9	33.7 ± 21.8	374.8 ± 189.3	130	0.0 ± 6.0	0.5 ± 0.2
2-*O*-desulfated MMWH_1	8771	1.6	80.7 ± 5.0	270.3 ± 42.5	38	10.0 ± 3.0	0.3 ± 0.4
2-*O*-desulfated MMWH_2	8776	1.5	91.9 ± 9.0	351.9 ± 43.4	23	0.0 ± 2.3	2.0 ± 2.0
2-*O*-desulfated MMWH_3	10,198	1.5	59.1 ± 16.5	487.9 ± 161.3	53	17.9 ± 2.1	0.5 ± 0.2
2-*O*-desulfated MMWH_4	11,190	1.4	68.0 ± 14.5	404.8 ± 119.4	33	7.5 ± 1.8	0.0 ± 0.0
2-*O*-desulfated glycol-split MMWH_1	7388	1.8	79.6 ± 5.4	893.1 ± 321.0	6	7.3 ± 4.5	0.4 ± 0.1
2-*O*-desulfated glycol-split MMWH_2	7037	1.5	84.2 ± 13.4	303.4 ± 46.2	5	3.6 ± 5.4	0.0 ± 0.0
ULMWH	4270	2.2	49.3 ± 6.0	236.3 ± 54.9	6	6.8 ± 9.2	0.2 ± 0.1
2-*O*-desulfated ULMWH_1	4150	1.6	140.9 ± 15.9	322.5 ± 27.5	ud.	20.0 ± 16.7	0.0 ± 0.0
2-*O*-desulfated ULMWH_2	4450	1.4	129.2 ± 13.1	262.5 ± 16.1	ud.	37.0 ± 9.9	0.2 ± 0.0
2-*O*-desulfated glycol-split ULMWH_1	4024	1.7	104.4 ± 6.0	192.4 ± 16.2	ud.	2.5 ± 2.3	0.0 ± 0.2
2-*O*-desulfated glycol-split ULMWH_2	3800	1.6	130.3 ± 15.5	200.5 ± 111.6	ud.	11.5 ± 8.0	0.0 ± 0.1
UH, commercial	13,000	1.9–2.0	9.4 ± 4.4	135.5 ± 12.8	197	0.0 ± 6.0	0.0 ± 0.0

^1^ Reported in vitro toxicity in human umbilical vein endothelial cell (HUVEC) culture (% of cell death) and hemolysis data (% of lysed RBCs) have been calculated with 2 mg heparin/mL. The numbers after two compounds with the same name indicate that those samples are replicates, made in order to check the reproducibility of the procedure and tested separately to corroborate if there were any differences in antimalarial or anticoagulant activity. n.d.: not determined, ud.: undetected.

**Table 3:** In vitro *P. falciparum* growth inhibition activity assay of the heparin-PQ conjugate.

Sample	Mw (Da)	IC50 (µM) ± SD
MMWH-PQ	13,945	1.16 ± 0.40
MMWH_1	11,000	3.1 ± 2.0 ^1^
PQ	259	5.20 ± 1.33

^1^ The molar concentration of MMWH was calculated considering a molecular weight of 11,000 kDa.

**Table 4:** Compilation of relevant data for heparin samples to be used in vivo.

Sample	IC50 (µg/mL ± SD)	aPTT (IU/mg)	In Vitro Toxicity ^1^ (% ± SD)	Hemolysis ^1^ (% ± SD)	In Vitro Toxicity ^2^
2-*O*-desulfated MMWH_2	91.95 ± 8.97	23	0.00 ± 2.34	1.99 ± 2.05	>320 mg/kg
ULMWH	49.31 ± 5.97	6	6.83 ± 9.18	0.21 ± 0.13	>320 mg/kg
2-*O*-desulfated glycol-split MMWH_1	79.60 ± 5.38	6	7.26 ± 4.55	0.36 ± 0.09	>750 mg/kg
2-*O*-desulfated glycol-split MMWH_2	84.20 ± 13.45	5	3.61 ± 5.42	0.00 ± 0.03	>750 mg/kg
2-*O*-desulfated glycol-split ULMWH_1	104.40 ± 6.03	0	2.49 ± 2.35	0.00 ± 0.22	>750 mg/kg

^1^ Reported in vitro toxicity in HUVEC culture (% of cell death) and hemolysis data (% of lysed red blood cells) have been calculated with 2 mg heparin/mL. ^2^ In vivo toxicity refers to the highest concentration tested that did not induce for 15 days after administration any acute or chronic effect in mice.

## Supplementary Materials

The following are available online at https://www.mdpi.com/1999-4923/12/9/825/s1, Figure S1: Partial ^1^H NMR spectra of heparin and its modifications; Figure S2: Partial ^13^C NMR spectra of heparin and its modifications; Figure S3: Partial ^13^C NMR, HSQC, and TOCSY spectra of 2-*O*-desulfated MMWH; Figure S4: Partial HSQC and TOCSY spectra of 2-*O*-desulfated glycol-split MMWH_1; Figure S5: Partial HSQC and TOCSY spectra of 2-*O*-desulfated glycol-split MMWH_2; Figure S6: Partial HSQC and TOCSY spectra of 2-*O*-desulfated glycol-split ULMWH_1; Figure S7: Partial HSQC spectra of ULMWH; Figure S8: ^1^H DOSY spectra of UH, MMWH, MMWH_1/2, ULMWH, and ULMWH_1.
