# Supplementary material for: Repurposing Heparin as Antimalarial: Evaluation of Multiple Modifications Toward In Vivo Application
Source: Pharmaceutics. 2020 Aug 29;12(9):825. doi: 10.3390/pharmaceutics12090825 (PMC7557421; doi:10.3390/pharmaceutics12090825)
Supplement: Supplementary file 1 [file pharmaceutics-12-00825-s001.pdf]

pharmaceutics

Supplementary Materials: Repurposing Heparin as Antimalarial: Evaluation of Multiple Modifications Toward In Vivo Application

Elena Lantero, Carlos Raúl Aláez-Versón, Pilar Romero, Teresa Sierra, and Xavier Fernàndez-Busquets

NMR characterization

When heparin is selectively *O*-desulfated at the C(2), the conversion of the IdoA2S residue into GalA or IdoA may occur [1]. The signals for H(1) and C(1) at 5.20 ppm and 102.2 ppm, respectively, characteristic of the IdoA2S residue disappear (black arrows in Figure S1 and S2). The formation of GalA is clearly observed in the HSQC spectrum of 2-*O*-desulfated MMWH, as indicated in Figure S3(b). For 2-*O*-desulfated glycol-split MMWH_1 (Figure S4), 2-*O*-desulfated glycol-split MMWH_2 (Figure S5) and 2-*O*-desulfated glycol-split ULMWH_1 (Figure S6), which have undergone subsequent glycol-splitting (see below), the IdoA2S residue is not observed.

Signals corresponding to the glycol-split in the GalA residue can be recognized in 2-*O*-desulfated glycol-split MMWH_1, 2-*O*-desulfated glycol-split MMWH_2 and 2-*O*-desulfated glycol-split ULMWH_1. The set of signals (^{1}H at 3.91 and ^{13}C at 62.9 ppm) which correlates with two protons in the TOCSY spectra at 4.25 and 4.64 ppm (red circles in Figures S4 and S5), is the signal pattern expected for a glycol-split uronic acid residue, with C(1) and C(4) bearing CH_2OH substituents, as previously reported in the literature [2].

For ULMWH, prepared by depolymerization by nitrous acid, the signal patterns observed in its ^{13}C and HSQC spectra (Figure S2 and S7) are consistent with the structure of dalteparin [3,4].

Finally, the application of DOSY NMR experiments has recently been proposed as a quick method for the determination of average molecular weight of heparins [5]. Here we have used this technique as a qualitative method to check the molecular weight of the samples (Figure S8). It is observed that the increase of the diffusion coefficient (D) corresponds to a decrease of molecular weight as expected according to the modifications carried out on UH.

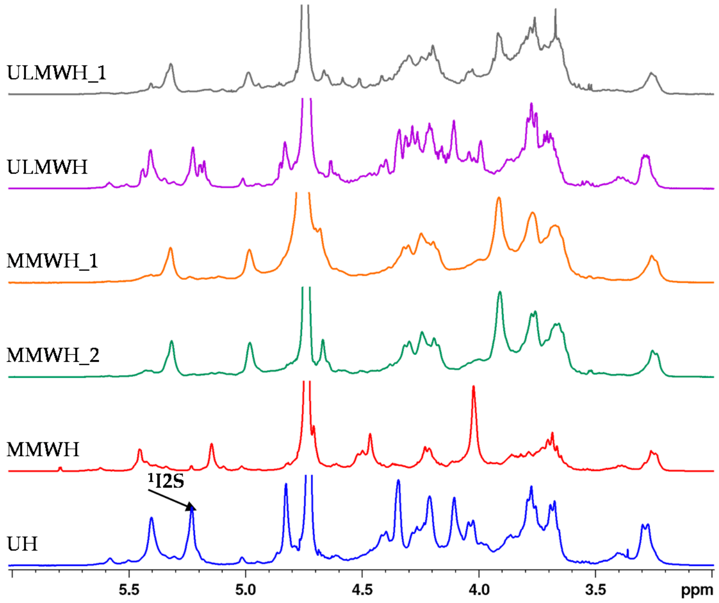

Figure S1. Partial ^{1}H NMR spectra of heparin and its modifications.

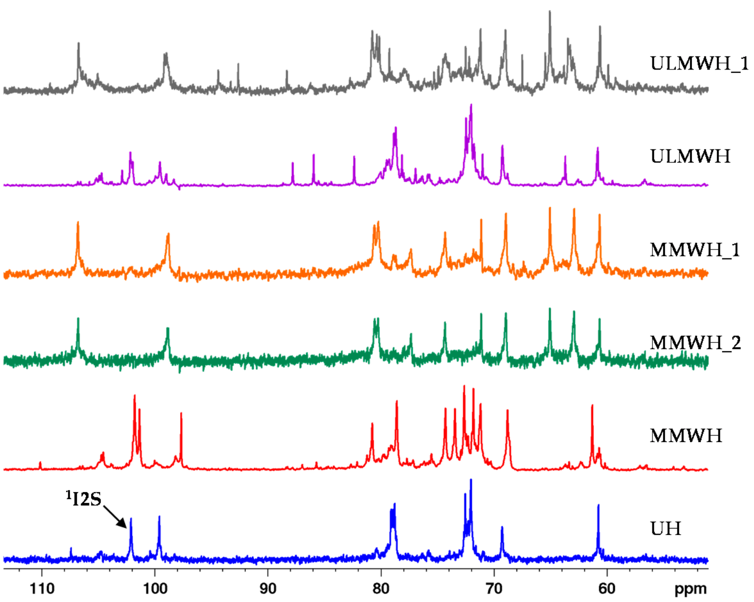

Figure S2. Partial ^{13}C NMR spectra of heparin and its modifications.

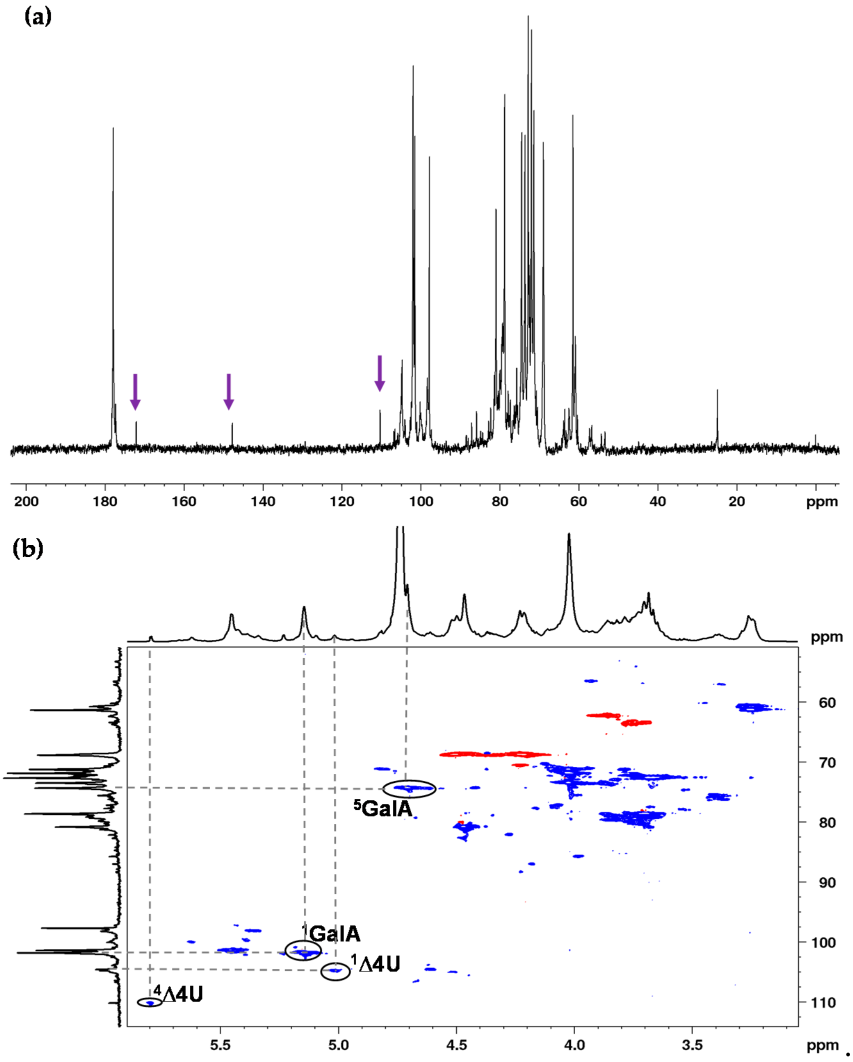

(c)

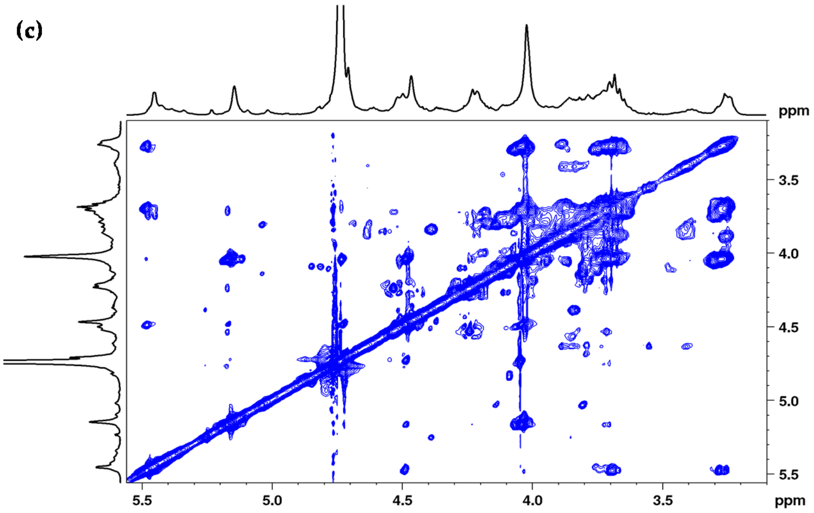

Figure S3. (**a**) Partial ^{13}C NMR spectrum between 200 and 10 ppm of 2-*O*-desulfated MMWH. There is some epoxide signal at 53-54 ppm and signals that correspond to β-elimination of uronic acid 2,5-insaturated ($^{4}\Delta U$) at 172, 147.7 and 110 ppm (purple arrows) (**b**) partial HSQC and (**c**) partial TOCSY spectra of 2-*O*-desulfated MMWH.

(a)

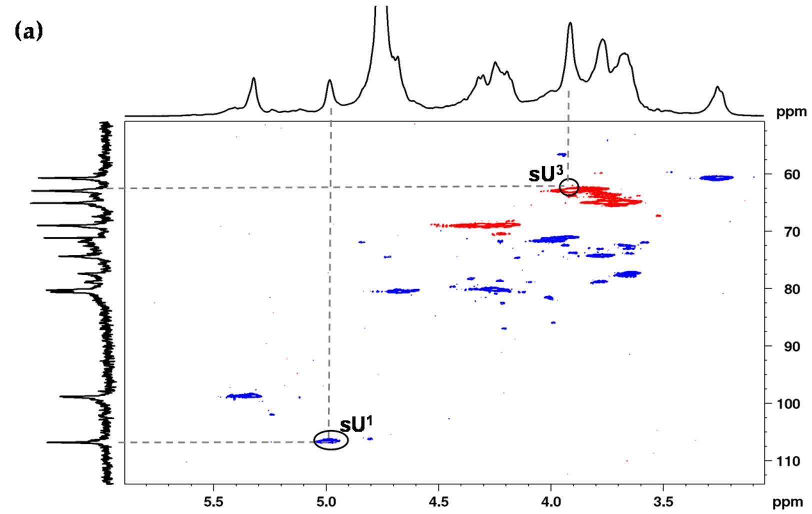

(b)

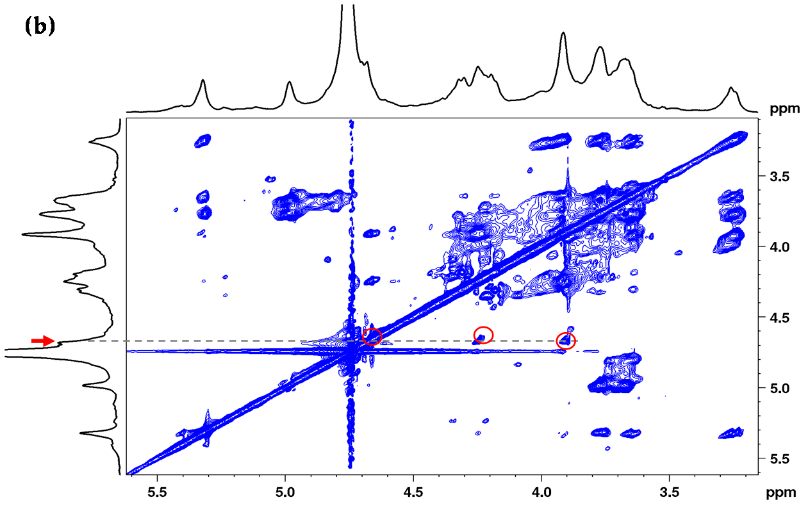

Figure S4. (**a**) Partial HSQC and (**b**) partial TOCSY spectra of 2-*O*-desulfated glycol-split MMWH_1.

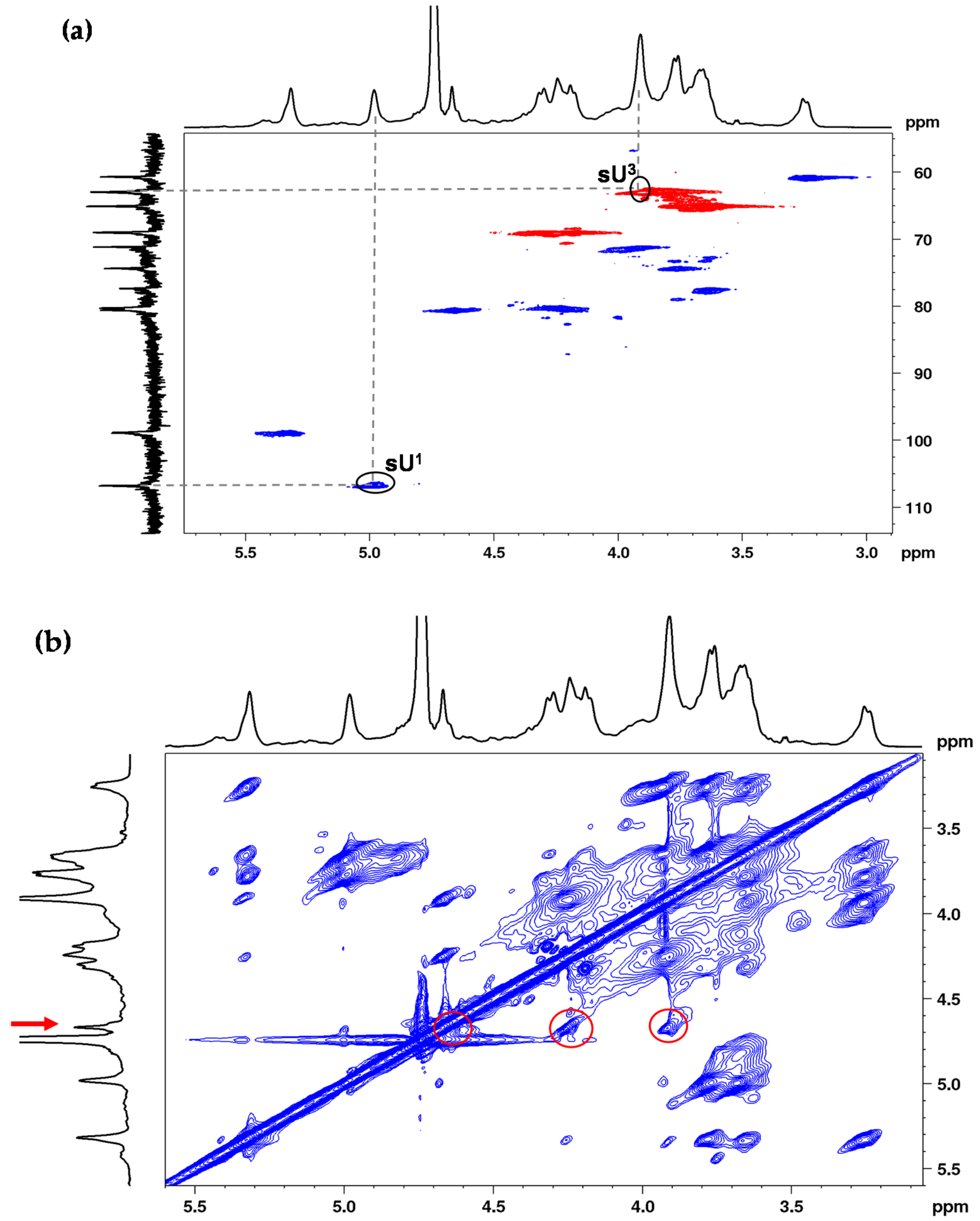

Figure S5. (**a**) Partial HSQC and (**b**) partial TOCSY spectra of 2-*O*-desulfated glycol-split MMWH_2.

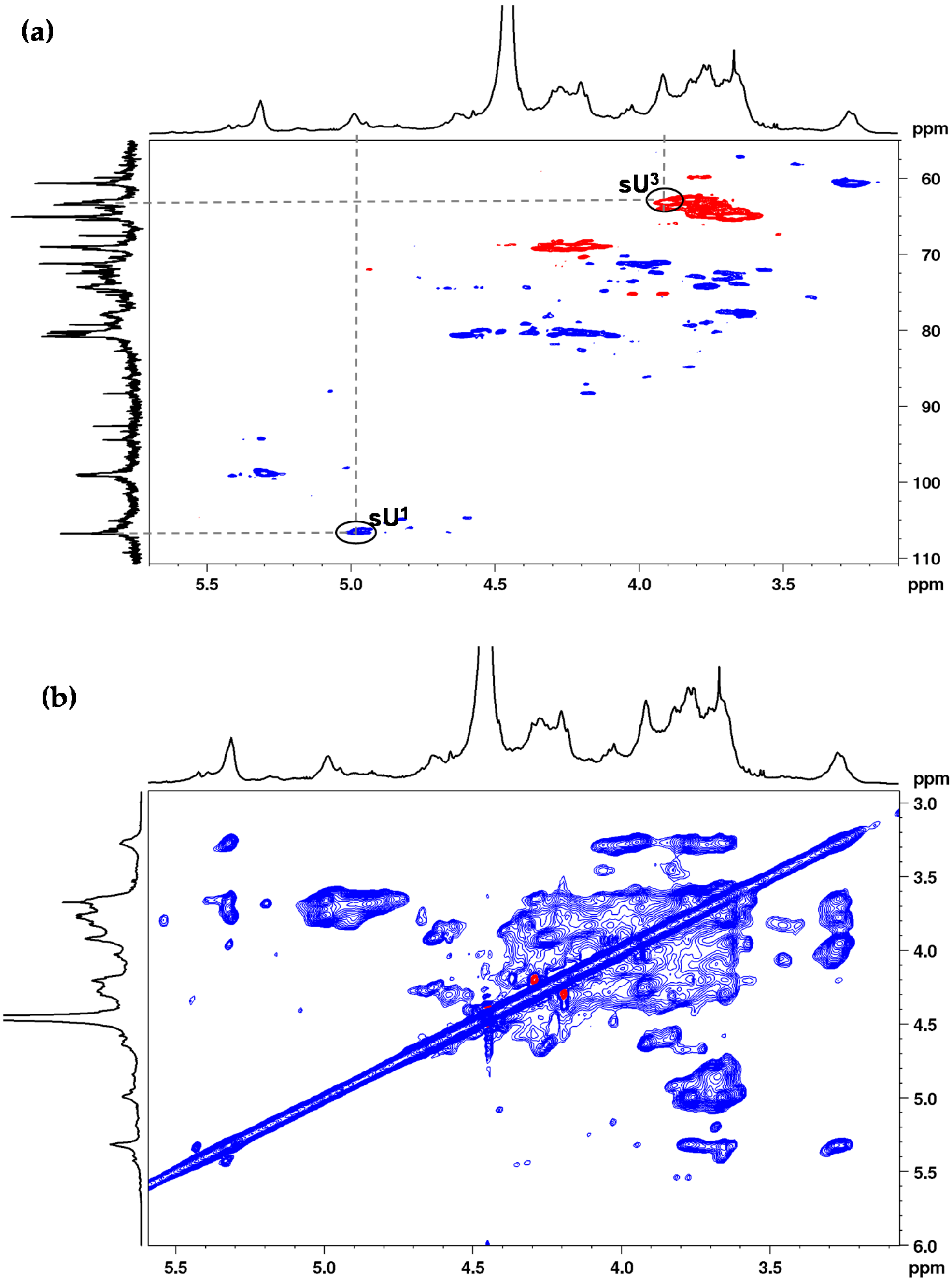

Figure S6. (**a**) Partial HSQC and (**b**) partial TOCSY spectra of 2-*O*-desulfated glycol-split ULMWH_1.

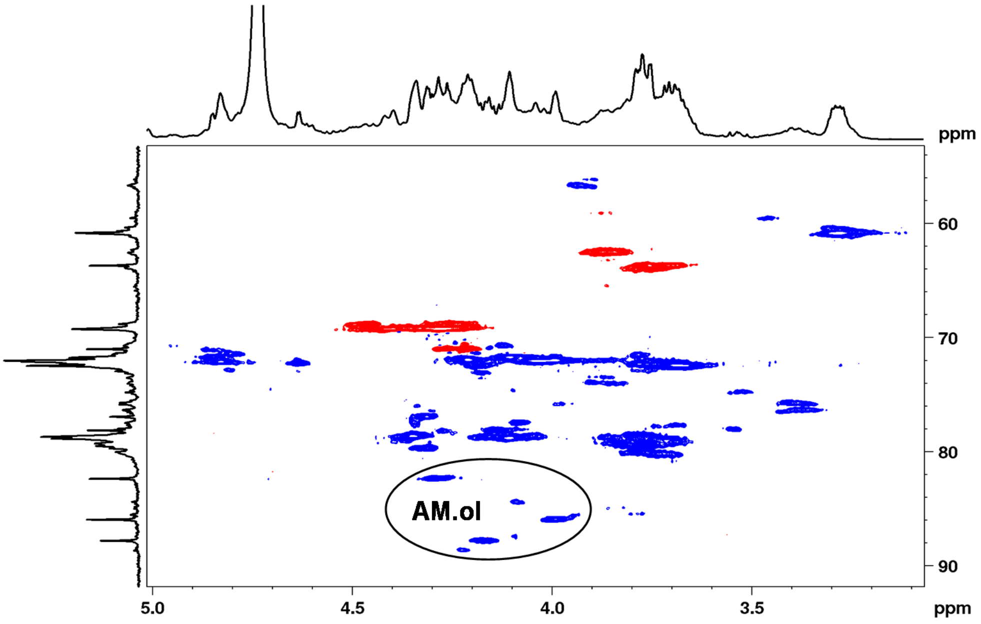

Figure S7. Partial HSQC spectra of ULMWH. Spectra in Figures S1 and S2 indicate that this molecule does not have 2-*O*-desulfation, and it relates with low molecular weight derivatives of heparin as dalteparin: 82.3, 85.9 y 87.8 ppm in ^{13}C correspond to C4, C2 and C5 of the 2,5-anhydro-D-mannitol residue (AM.ol), as observed in previous publications [3,4].

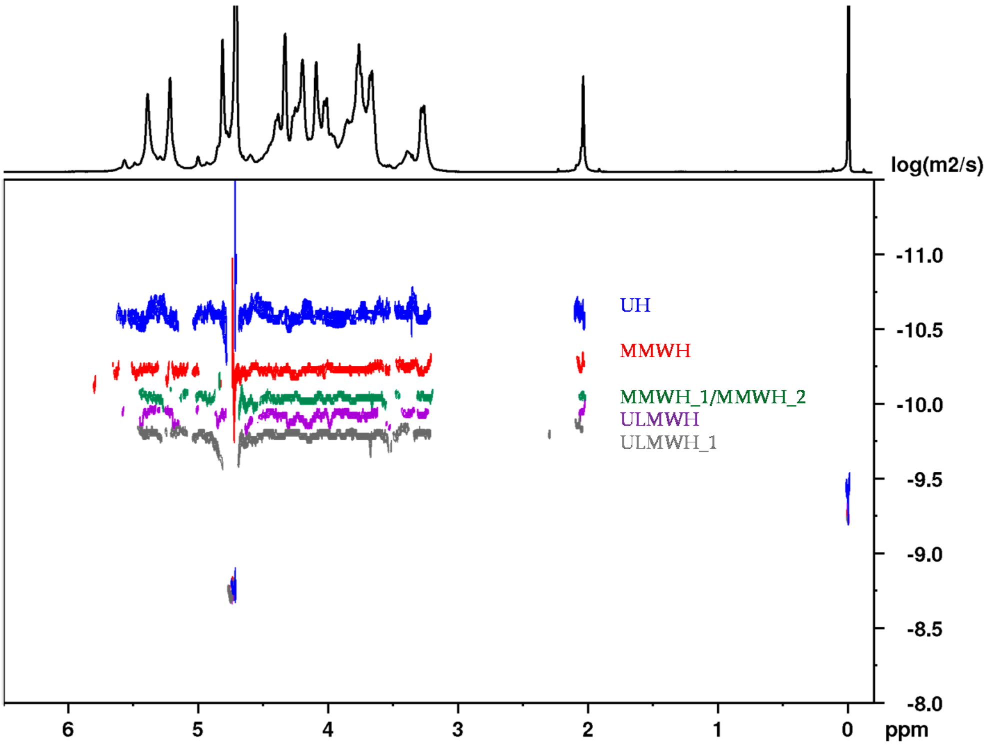

Figure S8. ^{1}H DOSY spectra of UH, MMWH, MMWH_1/2, ULMWH and ULMWH_1. The diffusion coefficients had been obtained relative to an internal standard (TPS) and their mobilities are inversely proportional to the molecular mass. The projection corresponds to UH.

References

1. Naggi, A.; Casu, B.; Perez, M.; Torri, G.; Cassinelli, G.; Penco, S.; Pisano, C.; Giannini, G.; Ishai-Michaeli, R.; Vlodavsky, I. Modulation of the heparanase-inhibiting activity of heparin through selective desulfation, graded *N*-acetylation, and glycol splitting. *J. Biol. Chem.* **2005,** *280* (13), 12103–12113.
2. Casu, B.; Guerrini, M.; Naggi, A.; Perez, M.; Torri, G.; Ribatti, D.; Carminati, P.; Giannini, G.; Penco, S.; Pisano, C.; Belleri, M.; Rusnati, M.; Presta, M. Short heparin sequences spaced by glycol-split uronate residues are antagonists of fibroblast growth factor 2 and angiogenesis inhibitors. *Biochemistry* **2002,** *41* (33), 10519–10528.
3. Guerrini, M.; Guglieri, S.; Naggi, A.; Sasisekharan, R.; Torri, G. Low molecular weight heparins: structural differentiation by bidimensional nuclear magnetic resonance spectroscopy. *Semin. Thromb. Hemost.* **2007,** *33* (5), 478–487.
4. Mauri, L.; Boccardi, G.; Torri, G.; Karfunkle, M.; Macchi, E.; Muzi, L.; Keire, D.; Guerrini, M. Qualification of HSQC methods for quantitative composition of heparin and low molecular weight heparins. *J. Pharm. Biomed. Anal.* **2017,** *136*, 92–105.
5. Monakhova, Y. B.; Diehl, B. W. K.; Do, T. X.; Schulze, M.; Witzleben, S. Novel method for the determination of average molecular weight of natural polymers based on 2D DOSY NMR and chemometrics: example of heparin. *J. Pharm. Biomed. Anal.* **2018,** *149*, 128–132.
